# 1889. The Epidemiology of Nontuberculosis Mycobacteria in a Single Urban Center

**DOI:** 10.1093/ofid/ofad500.1717

**Published:** 2023-11-27

**Authors:** Majd Alsoubani, Heidi Park, Tine Vindenes

**Affiliations:** Tufts Medical Center, Boston, Massachusetts; Tufts Medical Center, Boston, Massachusetts; Tufts Medical Center, Boston, Massachusetts

## Abstract

**Background:**

Non tuberculosis mycobacteria (NTM) are ubiquitous in the environment. The prevalence of NTM has increased worldwide, which is thought to be partly related to increased diagnostic testing but also possible aging populations, increased comorbid conditions and immunosuppression. NTM are considered rapidly emerging infections and often hard to treat. We aim to explore the true burden of NTM at Tufts Medical Center, a tertiary hospital serving a diverse patient population including a large immigrant Asian population

**Methods:**

This was a retrospective case review study done at Tufts Medical Center, a tertiary academic hospital in Boston, MA. We identified adult patients with an ICD-10 diagnosis of NTM between January 2012 and March 2022. Clinical and microbiologic data was collected from the medical record. Patients without microbiology data in our institution were excluded. Chi-square and Fisher’s exact tests were used for categorical variables, student T-test was used for age.

**Results:**

Out of 342 cases with an ICD-10 diagnosis of NTM, 197 patients met our inclusion criteria. In our sample, females represented 51.5% of cases. The majority of cases were white (48.2%) followed by Asian (42.6%). A third of patients with pulmonary NTM had a diagnosis of COPD and only 20% had documented past or current smoking history (Table 1). White patients were more likely to have extra pulmonary NTM compared to Asian patients (24 vs 5, p < 0.001). The most common site of NTM second to lung was skin and soft tissue (n=22, 10.9%). The most common NTM isolated from pulmonary site was Mycobacterium avium (n=85, 51.5%) while rapid growers [Mycobacterium chelonae (n=8, 25%), M.fortuitum (n=7,21.7%), and M. abscessus (n=7, 21.9%)] were the most common NTM isolated in extra pulmonary sites (Table 2).

**Figure:**
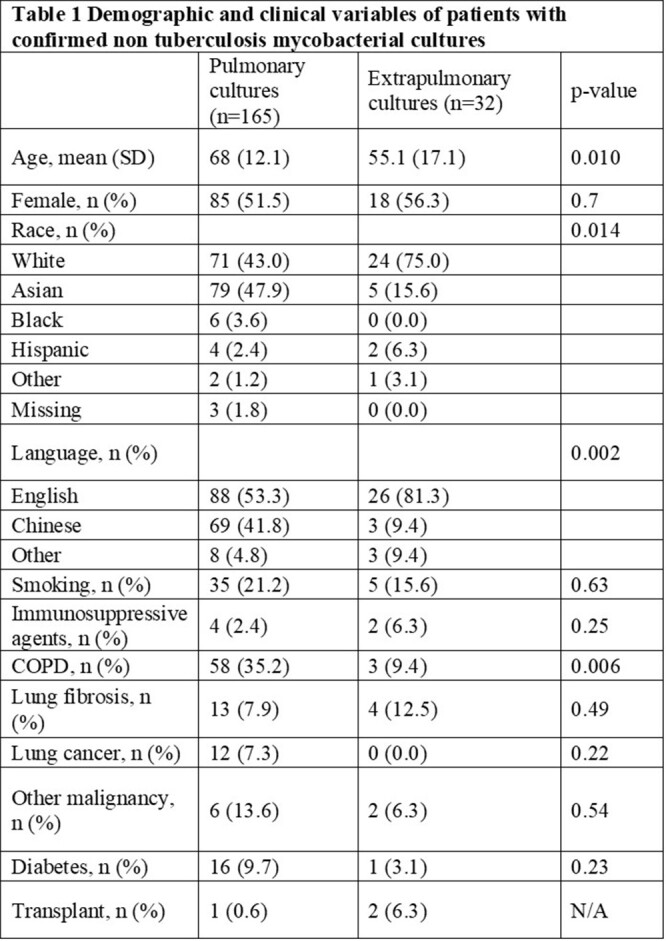

**Figure:**
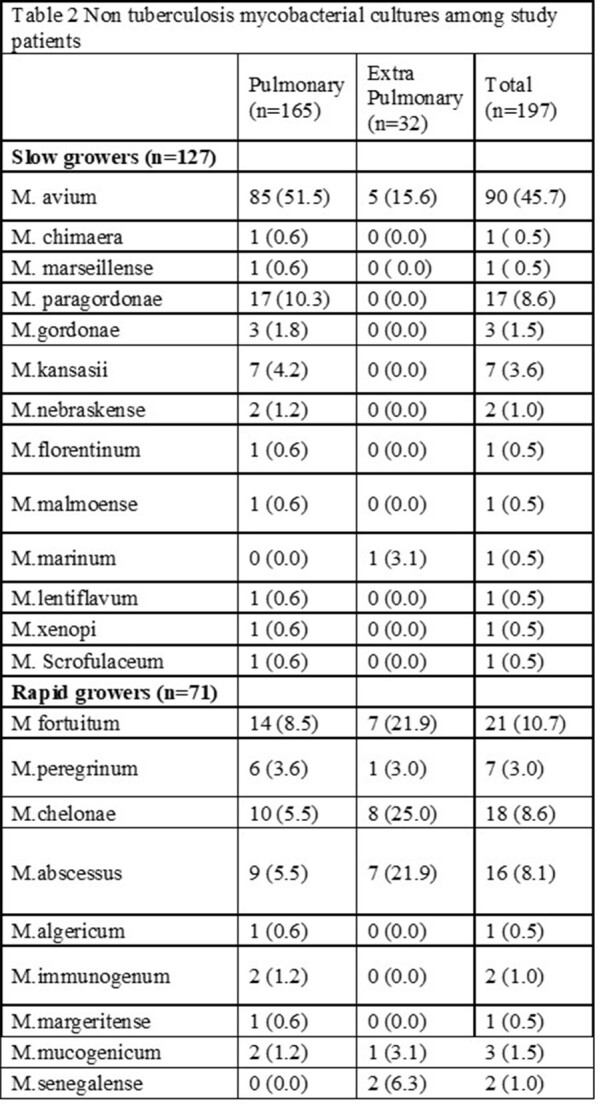

**Conclusion:**

The epidemiological review of NTM at our urban institution has improved the understanding of the population we serve. Our data show high percentage of Asians with pulmonary NTM cultures and suggest higher predominance of extrapulmonary NTM in Caucasians compared to Asians. The majority of the extrapulmonary NTM were rapid growers. Larger epidemiologic studies and surveillance data are needed to better understand NTM in various environments and hosts.

**Disclosures:**

**All Authors**: No reported disclosures

